# 660–808 nm simultaneous irradiation enhance keratinocyte migration and change the keratin expression: An *in vitro* study

**DOI:** 10.1111/php.14124

**Published:** 2025-05-27

**Authors:** Renan Carrasco Zuffo, Anaeliza Figueiredo dos Santos, Luciana Corrêa

**Affiliations:** ^1^ School of Dentistry University of São Paulo São Paulo Brazil

**Keywords:** double laser irradiation, keratinocytes culture, keratins, red and infrared lasers

## Abstract

This in vitro study aimed to determine if simultaneous irradiation with 660 and 808 nm wavelengths enhances keratinocyte migration and alters keratin expression. Keratinocytes were exposed to either 660 nm or 808 nm irradiation alone, as well as to both wavelengths simultaneously using a laser device with the same energy parameters (2 J, 22.22 J/cm^2^). Cell viability, migration, and keratin expression (K1, K10, K5, and K14) were assessed in a scratch model assay. After 24 h of PBM, the simultaneous group showed higher cell viability compared with the control and the irradiated groups with one wavelength (660 and 808 nm groups). Simultaneous irradiation also resulted in a smaller scratch area compared with the control and 660 nm groups. The frequency of cells positive for K1/K10 at the wound border was lower after dual irradiation, while cells positive for K5/K14 at the wound periphery were more frequent after simultaneous irradiation. These results suggest a potential increase in the population of less‐differentiated keratinocytes following 660–808 nm dual emission. In conclusion, combined irradiation improved cell viability and migration, potentially leading to a shift in keratinocyte differentiation. This dual‐wavelength effect may help stimulate the reepithelization process in the tissue repair.

## Graphical abstract

Exposure to both 660 and 808 nm wavelengths at the same time induced intense keratinocyte migration and altered keratin expression, indicating a potential enhancement of a subset of less‐differentiated keratinocytes.

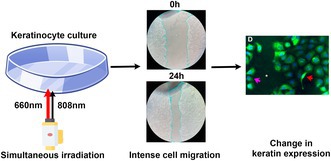

**Abbreviations**
K: keratin
PBM: photobiomodulation

## INTRODUCTION

Photobiomodulation (PBM) is commonly used to modulate inflammation, reduce pain, and promote tissue repair. In certain clinical scenarios, dual simultaneous irradiation with 660 and 808 nm wavelengths has been utilized for analgesic purposes.^1^ The combined action on various chromophores targeted by these wavelengths and their impact on different tissue layers are key features of simultaneous irradiation that may help manage inflammation and enhance the healing process.

During the clinical application of PBM, the epithelium is the tissue layer that is most likely to directly interact with a dual emission of two wavelengths simultaneously. This observation leads to the hypothesis that one of the primary effects of this dual emission on tissue repair could be on the reepithelialization process. However, the impact of this dual emission on keratinocytes is not well understood. A study conducted by our group in vitro demonstrated that simultaneous irradiation with 660 and 808 nm wavelengths caused an imbalance in antioxidative markers in primary gingival keratinocytes and did not improve cell viability, particularly after 20 s of irradiation.^2^ Further research is needed to fully comprehend the biological significance of this effect, particularly in relation to other aspects of the reepithelialization process.

Epithelial regeneration involves the proliferation, differentiation, and migration of keratinocytes, which is associated with the expression of specific keratins in the cytoskeleton.^3^ Keratins are intermediate filaments that make up the cytoskeleton and are involved in cell–cell and cell‐extracellular matrix interactions through desmosomes and hemidesmosomes, contributing to the integrity and mechanical stability of both individual epithelial cells and epithelial tissues.^4^

Keratins are found in pairs, with low molecular weight acidic keratins (type I, numbered 9 to 19) combining with high molecular weight basic or neutral keratins (type II, numbered 1 to 8) to form heterodimers.^4^ The K5/K14 pair is highly expressed in the undifferentiated basal epithelial layer but less so in suprabasal cell layers undergoing differentiation.^5^ In these layers, the K1/K10 pair is more expressed, indicating terminal cell differentiation, particularly in cutaneous and oral epithelium.^6^ Both K5/K14 and K1/K10 pairs have been investigated in normal and pathological skin and oral mucosa epithelium as markers of keratinocyte differentiation and migratory potential.^7^, ^8^, ^9^

The function of keratins in cell migration remains unclear. It is thought that they play a role in providing a balancing force during migratory movement, which is triggered and driven by the actin‐myosin system.^3^ Keratins contribute to cell elasticity while also reinforcing cells to prevent breakage and withstand tension loads.^3^ In vitro experiments have shown that the pair K5/K14, for example, provides greater resilience to basal cells of stratified epithelia,^10^ while also contributing to proliferation and migration to the upper layers.^11^ On the contrary, the pair K1/K10 is associated with the cessation of this migratory movement, while the cell reaches its highest stage of differentiation.^3^

There is a general consensus that exposure to red spectrum irradiation (661 to 660 nm) enhances the migration of skin keratinocytes,^12^, ^13^, ^14^ particularly at higher power densities (10 to 15 mW/cm^2^).^12^ Research on the impact of PBM on keratin expression is limited, with most studies focusing on epithelial regeneration in in vivo models. These studies have demonstrated a significant increase in K14 expression in epithelial projections during wound healing following PBM,^15^, ^16^, ^17^ as well as an upregulation of K10 expression upon complete reepithelialization.^15^, ^18^ However, there is a lack of scientific literature investigating keratin expression following dual 660–808 nm emission.

The aim of this study is to examine how simultaneous irradiation at wavelengths of 660–808 nm affects keratinocyte migration in vitro, as well as its impact on the expression of K1/K10 and K5/K14 during cell migration. Given the enhanced potential of simultaneous irradiation to alter cell structure and baseline metabolism,^2^ we hypothesize that this type of irradiation can increase the rate of cell migration and subsequently alter keratin expression during this process.

## MATERIALS AND METHODS

### Ethical aspects

The Ethics Committee on Human Research of our institution previously approved the methodology of this study (CAAE 08341019.0.0000.0075).

### Cell culture and experimental groups

Keratinocytes (HaCat) were cultured in Dulbecco's Modified Eagle Medium (DMEM) (Sigma Chemical Co., St. Louis, MO, USA), supplemented with 10% fetal bovine serum (Gibco ‐ Invitrogen, Carlsbad, CA, USA), 1% antibiotic/antimycotic (Gibco), and 1% L‐glutamine in a T175 flask. The cells were maintained in a 5% CO_2_ incubator at 37°C until reaching 80% confluence. The study included the following groups: Control—without any treatment and maintained under the same conditions as the other groups; 660 nm—cells were irradiated with a 660 nm laser emission; 808 nm—cells were irradiated with an 808 nm laser emission; and Simultaneous—cells were irradiated with 660 and 808 nm emitted at the same time (simultaneous irradiation).

### Photobiomodulation technique

**FIGURE 1:**
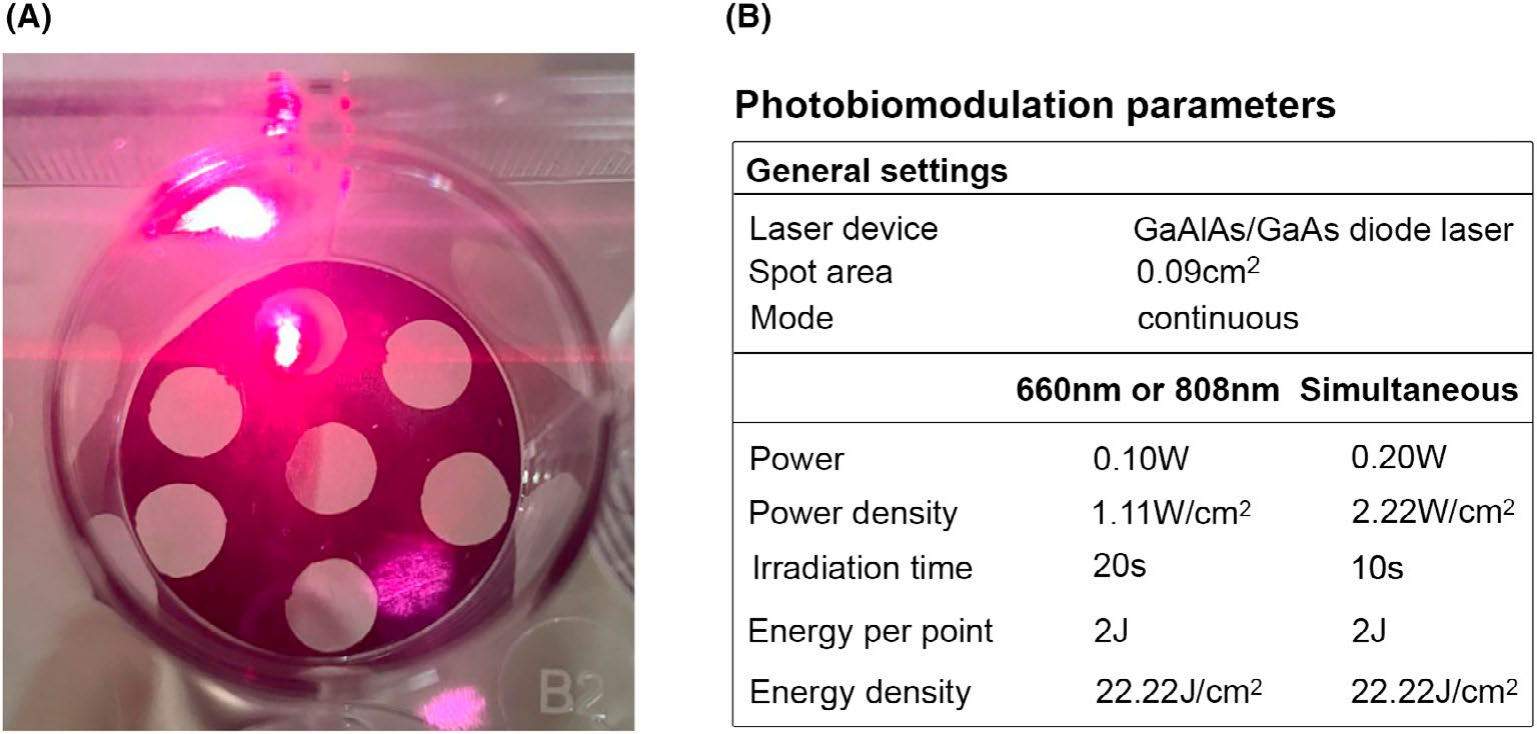
(A) Photobiomodulation was conducted in cell culture. The laser probe was placed perpendicular to the plate base, making contact with an orifice marked by a black adhesive to prevent overlapping irradiation in the wells. (B) Light parameters employed in the photobiomodulation groups.

The cells were exposed to radiation using a GaAlAs/GaAs diode laser device with single wavelengths of 660 and 808 nm, as well as a simultaneous mode of the two wavelengths (LaserTherapy EC, DMC Equipamentos, São Carlos, Brazil). For the irradiation process, the cells were placed in a dish with a black adhesive background that had orifices matching the diameter of the laser probe. The probe was positioned perpendicularly in the orifice below the dish (Figure 1A). Two sessions of PBM were conducted with a 3‐h interval between them, using standardized parameters (Figure 1B).

### Cell viability

To assess cell viability, keratinocytes (10^4^) were treated with PBM in a 96‐well plate with wells having translucent backgrounds and the space between them being black. The black background was selected to reduce photon dispersion in the plate. PBM was carried out following the methods and parameters previously described. After the final PBM session, cell viability was determined using a commercial kit (Cell Titer 96 kit from Promega, Madison, Wisconsin, USA) that detects the conversion of tetrazolium salt to formazan, leading to a pink color in the solution, indicating active cell metabolism. The formation of formazan salt was measured with a spectrophotometer (ELX 800 Biotekinstruments Inc., Winooski, VT, USA) using a 490 nm filter. The cell viability assay was carried out in triplicate in two separate experiments, resulting in a total of six repetitions. The experimental repetitions yielded a mean value, which was then normalized by dividing it by the control mean values and multiplying by 100. The final result was expressed as a percentage.

### Scratch assay

After 24 h of culturing keratinocytes (10^5^ cells) in a 12‐well plate, a horizontal and continuous scratch was made in the cell monolayer using a 200 μL pipette tip. The scratch size and direction were standardized by the same operator, who used a horizontal line in the well background as a reference. Immediately after creating the scratch (“wound”), the defect image was captured at 10x original magnification using a digital camera and an inverted microscope (Axio Vert®, Carl Zeiss, Oberkochen, Germany). The plate's position in the microscope was adjusted using the horizontal line in the well background. Subsequently, PBM was performed according to the assigned groups. The wound image was taken 24 and 48 h after the last irradiation. Since the wound was fully closed at 48 h, the wound area was calculated only for the 24‐h time point. The wound area was measured using a plugin specifically designed for scratch assay analysis^19^ within the ImageJ software.^20^ The final percentage of wound area was calculated using the formula: (wound area at 24 h/wound area at baseline) × 100. Additionally, the homogeneity of the wound was assessed by determining the standard deviation of the mean width. The scratch assay was conducted in triplicate across two separate experiments, resulting in a total of six replicates.

### Keratins expressions

Keratin expression was assessed through immunofluorescence assay during cell migration. A scratch assay was conducted with keratinocytes plated on coverslips following the same procedures as described previously. After 24 h of the scratch and PBM, cells were fixed in 4% paraformaldehyde and subjected to immunofluorescence analysis. Primary antibodies (Abcam, Cambridge, UK) against K1 (rabbit, polyclonal, 1:100), K10 (mouse, DE‐K10 clone, 1:100), K5 (rabbit, EP1601Y clone, 1:50), and K14 (mouse, SP53 clone, 1:50) were incubated overnight at 4°C. The secondary antibody used was a fluorescein isothiocyanate (FITC) goat anti‐mouse antibody, incubated for 60 min at room temperature. Nuclear staining was performed with Fluoreshield Mounting Medium containing 4',6‐diamidino‐2‐phenylindole (DAPI) (Abcam). Keratin expression levels were examined using a fluorescence microscope (Axioscope A1, Carl Zeiss, Oberkochen, Germany) with 470 and 528 nm filters. The percentage of positive cells was determined through manual counting using a cell counter plugin in ImageJ software. Fields stained with FITC and DAPI at ×400 original magnification, containing cells at the periphery and border of the wound, were digitized. An operator, blinded to the groups, merged the FITC and DAPI images and manually counted the total number of nuclei and keratin‐positive cells in each field. A minimum of 500 nuclei were counted on each coverslip. The total number of K1‐positive cells was combined with the number of K10‐positive cells, along with the total number of nuclei counted for each keratin, to calculate the percentage of K1/10‐positive cells. The same method was used for the K5/K14 pair. Each group was tested in duplicate for the assay.

### Statistical analyses

Data from the cell viability and scratch assays were analyzed using parametric methods and presented as mean ± standard deviation. Group comparisons were conducted using ANOVA followed by Tukey's test. The frequency of keratin‐positive cells was compared between groups using the chi‐square test with Yates' correction. Statistical significance was defined as *p* < 0.05.

## RESULTS

### Simultaneous irradiation increases cell viability

**FIGURE 2:**
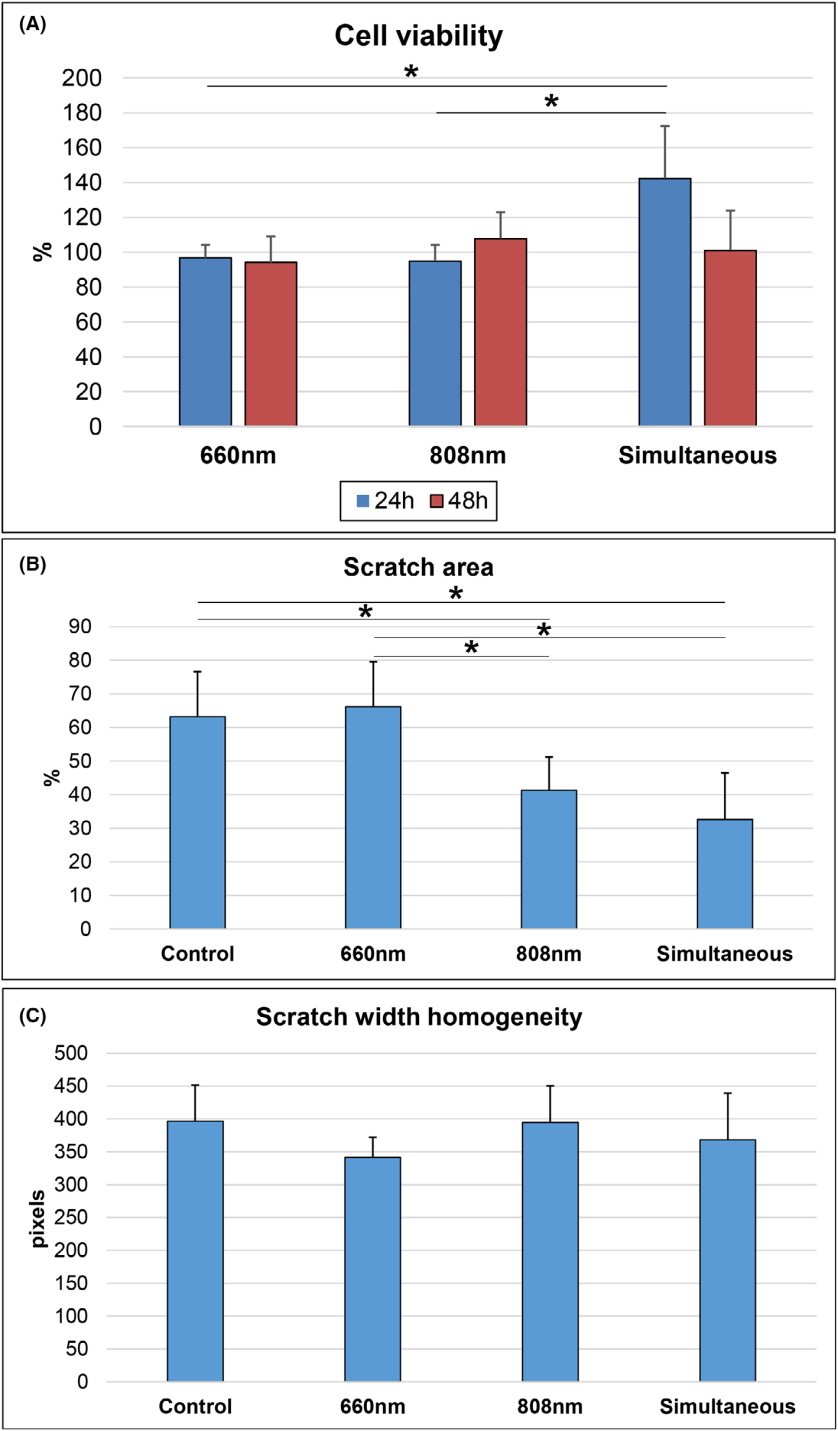
(A) Mean (±standard deviation) percentage of cell viability in the photobiomodulation groups (660, 808, and 660–808 nm simultaneous irradiation) after 24 and 48 h of the photobiomodulation sessions. The values are expressed in percentage because they were normalized by the control. (B) Mean (±standard deviation) wound area in the control and photobiomodulation groups after 24 h of the last irradiation session. The values were normalized to those obtained at baseline (before the irradiation) in each group. (C) Mean (±standard deviation) wound width standard deviation in the control and photobiomodulation groups after 24 h of the last irradiation session. *p < 0.05. *p* value by Tukey test.

After 24 h of the PBM treatment, the Simultaneous group showed a stimulating effect on cell viability compared with the control group. Cell viability was also significantly higher compared with the 660 nm (*p* < 0.05) and 808 nm (*p* < 0.05) groups (Figure 2A). This stimulating effect disappeared after 48 h, returning to levels similar to the control group. At this time point, only the 808 nm wavelength led to an increase in cell viability, but the difference was not statistically significant compared with the other groups.

### Simultaneous irradiation induces a lower scratch area

**FIGURE 3:**
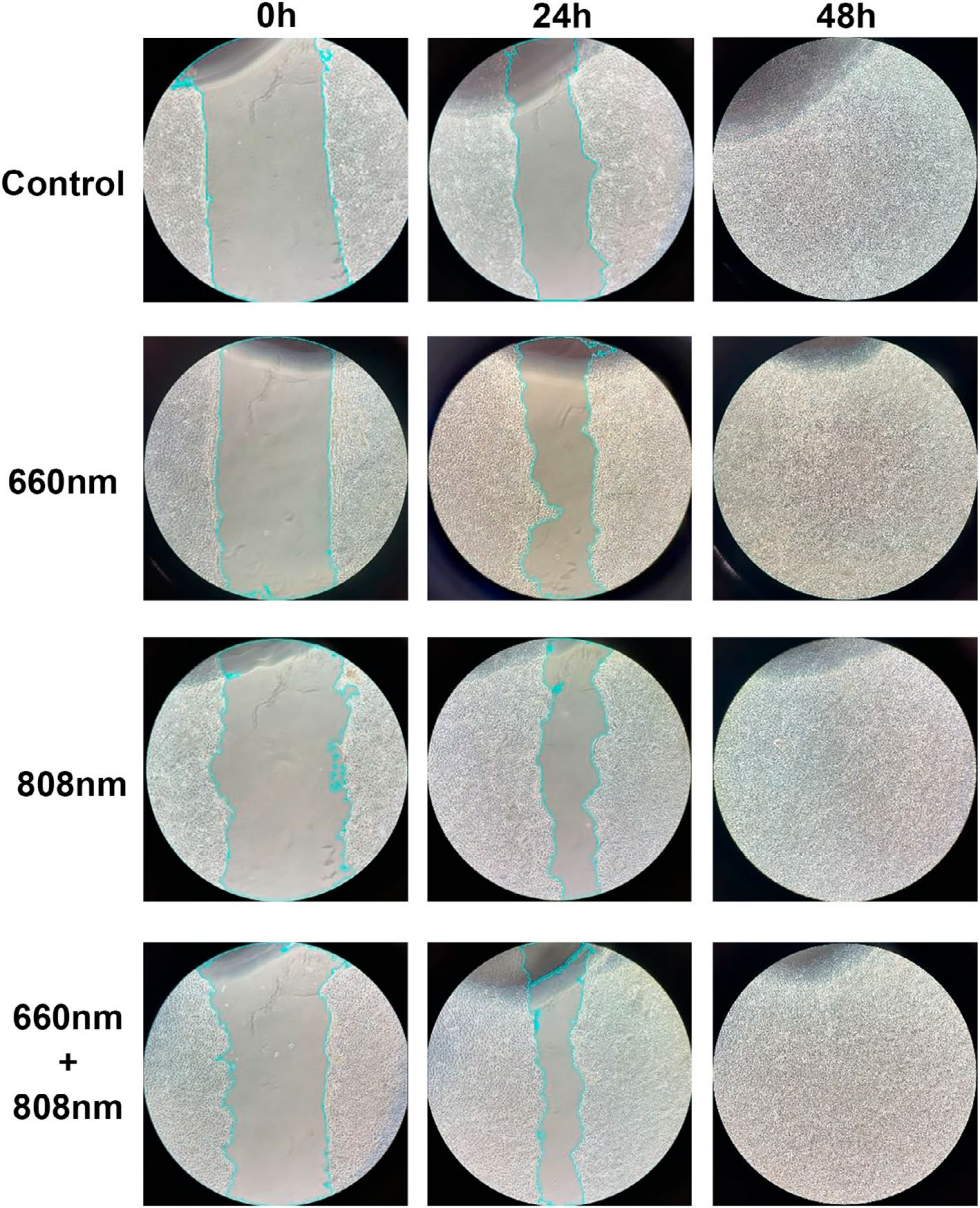
Representative images of the wound boundaries in the scratch assay for the control and photobiomodulation groups (660, 808, and 660–808 nm simultaneous irradiation). The images were taken immediately after creating the wound (0 h) and after 24 and 48 h following two consecutive photobiomodulation sessions.

By 48 h, the cell monolayer defect was fully healed in all groups (Figure 3). The Simultaneous and 808 nm groups had the smallest scratch area after 24 h of PBM sessions, with significant differences compared with the Control (*p* < 0.05 for all comparisons) and 660 nm groups (*p* < 0.05 for all comparisons) (Figure 2B). The standard deviation of the wound width was similar across all groups after 24 h of irradiation, indicating consistent wound homogeneity (Figure 2C).

### Keratin was expressed with different patterns in the scratch border

**FIGURE 4:**
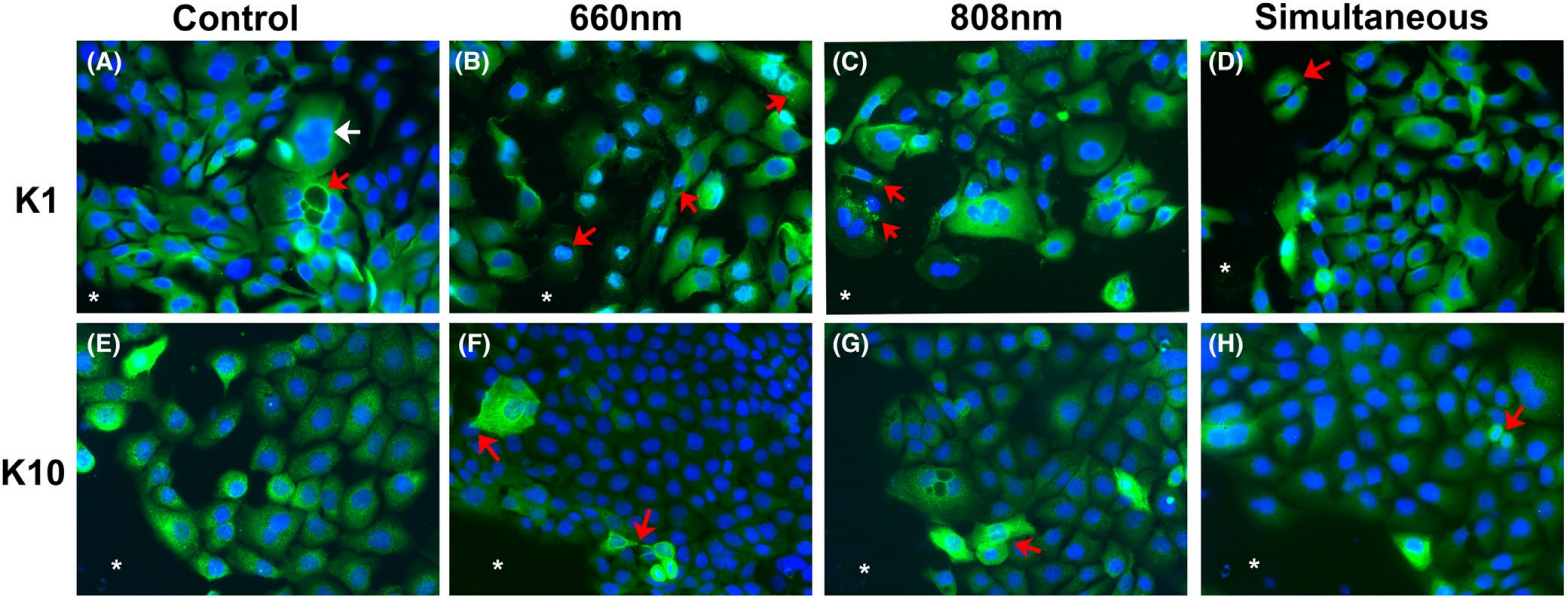
Representative K1 (A–D) and K10‐positive cells (E–H) in the wound border of each group. Different patterns of keratin expression were observed in all experimental repetitions. (A) K1 expression was homogeneous in the keratinocytes with typical size or increased size (white arrow); some cells showed intracytoplasmic vesicles positive for K1 (red arrow). (B) Perinuclear K1 expression was observed in some cells (arrow). (C) K1 was detected as immunofluorescent aggregates in other cells (arrow). (D) Most migrating cells in the wound space were K1‐positive (arrow). (E) K10 was also homogenously expressed in the cytoplasm of keratinocytes. (F) Keratinocytes at the wound border showed cytoplasmic projections positive for K10, indicating a migration direction (arrows). (G) Cells at the wound border showed strong K10 positivity (arrows). (H) Cells with reduced size showed also a strong K10 positivity (arrow). *Wound space. Blue color represents DAPI staining, and green color represents FITC staining. Original magnification ×400.

In all groups, K1 was mainly expressed with a consistent distribution in the cytoplasm at a similar intensity (Figure 4A–D). Some cells near the scratch border in the irradiated groups showed a halo of K1 filaments around the nucleus (Figure 4B) or fluorescent aggregates in the cytoplasm (Figure 4C). The migrating cells near the wound area were mostly positive for K1 (Figure 4D).

K10 exhibited a range of expression levels in the cytoplasm of most cells, with some showing mild expression and others showing intense expression (Figure 4E–H). Cells with intense K10 expression were mainly found near the scratch border. The intense expression indicated the presence of filaments arranged spatially with projections that indicated the direction of cell migration (Figure 4F, G). Additionally, some K10‐positive cells displayed a rounded nucleus and decreased cytoplasm, suggesting an undifferentiated keratinocyte morphology (Figure 4H).

**FIGURE 5:**
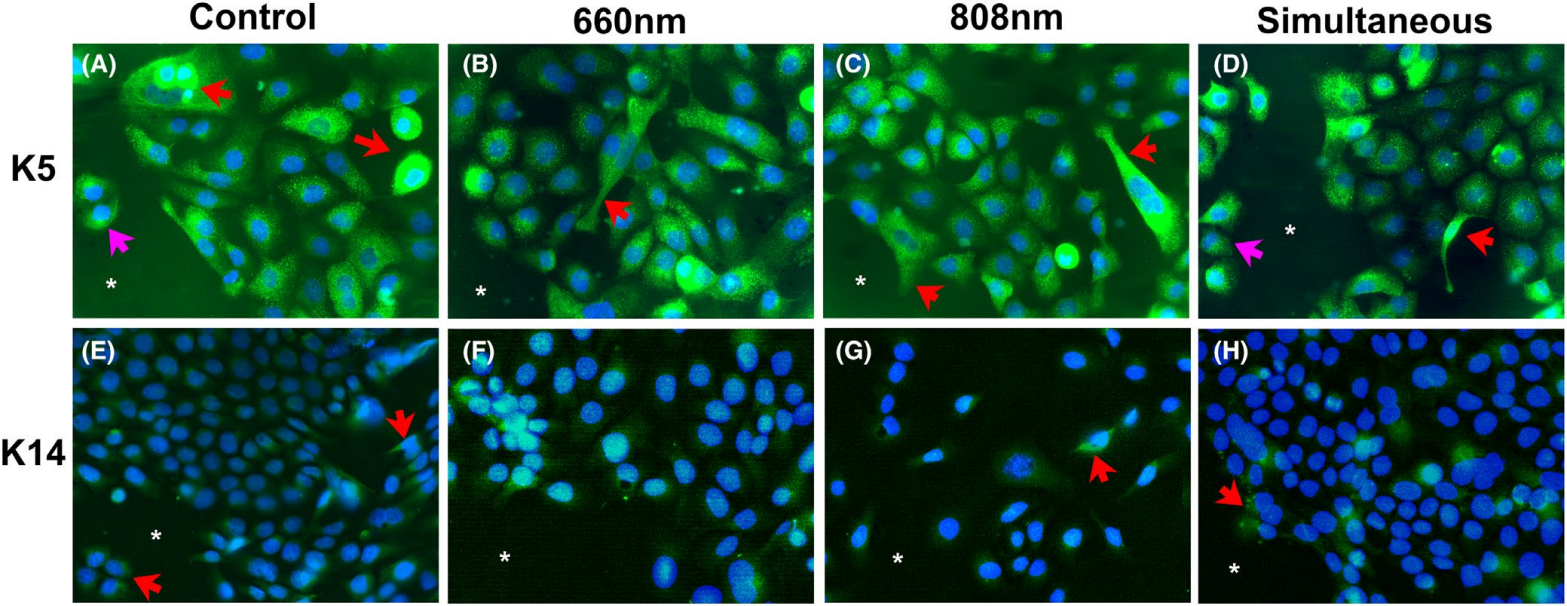
Representative K5‐ (A–D) and K14‐positive cells (E–H) at the wound border in each group. Different patterns of keratin expression were observed in all the experimental replicates and did not show a consistent trend within each group. (A) Moderate to strong K5 expression (arrow); some migrating keratinocytes in the wound area were K5‐positive (pink arrow). (B, C) K5 expression revealed cytoplasmatic projections in elongated cells, suggesting a migration movement (arrows). (D) K5‐positive cells in the wound space were observed in clusters (pink arrow); elongated morphology suggestive of a mesenchymal phenotype was also seen in K5‐positive cells (red arrow). (E–G): Mild cytoplasmic expression of K14 (arrows) with a granular pattern. H: Strong K14 expression in cytoplasmic aggregates in some cells (arrow). *Wound space. Blue color represents DAPI staining, and green color represents FITC staining. Original magnification ×400.

K5 was detected in the cytoplasm with a granular pattern (Figure 5A–D). Intense expression was observed in certain cells, particularly in the layers adjacent to the cell‐free space upon border analysis (Figure 5A–D). Elongated K5‐positive cells with cytoplasmic projections indicative of migration were also observed (Figure 5B–D). Additionally, K5‐positive cells were present in the scratch space (Figure 5A–D).

The frequency of K14‐positive cells was lower and had a less intense staining compared with the other keratins in all the groups (Figure 5E–H). Cell clusters near the cell‐free space showed rare K‐14 positivity (Figure 5E). K14‐positive aggregates were observed in the cytoplasm of some cells (Figure 5H).

### K1/K10 were the predominant keratins in all groups during cell migration

**FIGURE 6:**
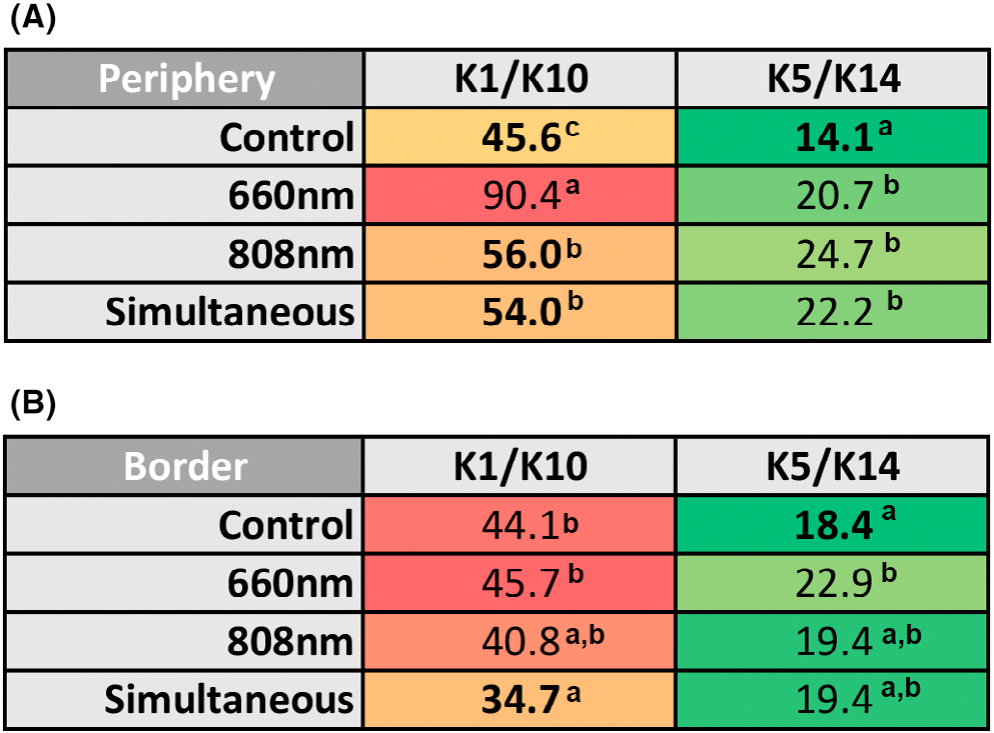
Frequency of positive cells for K1/K10 and K5/K14 pairs detected in the periphery (A) and in the border (B) of the wounds in both the control and the photobiomodulation groups (660, 808, and 660–808 nm simultaneous irradiation). Different letters indicate a significant difference between the groups as determined by the chi‐square test (*p* values are shown in the text).

Figure 6 illustrates the frequency of positive cells for each keratin. In both the scratch periphery and border, the expression of the K1/K10 pair was higher than that of the K5/K14 pair. In the periphery, the 660 nm group had the highest frequency of K1/K10‐positive cells, with significant differences compared with the Control and other irradiated groups (*p* < 0.001 for all comparisons). The 808 nm (*p* = 0.030) and Simultaneous (*p* = 0.048) groups also had a higher frequency of K1/K10‐positive cells in the scratch periphery compared with the Control. In the border, the Simultaneous group showed reduced K1/K10 expression, with a significant difference compared with the Control (*p* = 0.006) and 660 nm (*p* < 0.001). Concerning the K5/K14‐positive cells, in the scratch periphery of the Control group, the expression of this keratin pair was notably lower than in all the irradiated groups (*p* < 0.001 for all comparisons). This trend continued in the scratch border, with significant differences observed between the Control group and the 660 nm group (*p* = 0.037).

Overall, the findings indicated that the Simultaneous group showed a tendency to have fewer K1/K10 positive cells in the wound border compared with the Control and 660 nm groups. On the contrary, simultaneous irradiation did not significantly increase the expression of K5/K14 in this wound region. The 808 nm group exhibited a similar pattern. The 660 nm group had the highest frequency of cells positive for K1/K10 (mostly in the periphery) and K5/K14 (mostly in the border) among the irradiated groups.

## DISCUSSION

The primary result of the study indicated that the simultaneous irradiation of 660 and 808 nm wavelengths increased cell migration more effectively than using either 660 nm or 808 nm alone. This enhancement in cell migration was associated with alterations in keratin expression.

Initially, we conducted a cell viability assay to confirm that the light parameters utilized in the Simultaneous group (200 mW, 2.2 W/cm^2^) did not induce phototoxic effects on the keratinocytes, which could potentially impact the migration assay. The Simultaneous group exhibited a higher percentage of viable cells after 24 h of irradiation, indicating that the parameters employed were not inhibitory. This outcome contrasted with a previous study conducted by our team using primary gingival keratinocytes, where simultaneous irradiation led to a decrease in cell viability under the same light parameters.^2^ It is likely that the immortalized keratinocytes (HaCat) utilized in the current study responded differently to the two wavelengths, promoting cell replication compared with primary cell cultures.^21^

Simultaneous exposure to 660 and 808 nm wavelengths exhibited a greater potential for wound closure, consistent with the stimulatory effects observed in the cell viability assay. Treatment with 808 nm alone also resulted in a reduction in wound area comparable to that seen with simultaneous irradiation. There is limited existing literature on the combined effect of 660 and 808 nm wavelengths on cell migration, with previous studies focusing on the stimulatory effects of red‐light spectrum PBM on keratinocyte migration^12^, ^13^, ^14^ using 0.18, 3, 6, 7 J/cm^2^, respectively. In our study, irradiation with 660 nm did not significantly reduce wound area compared with the control or other PBM groups. This discrepancy may be attributed to differences in parameters, particularly the higher energy density (22.2 J/cm^2^) used in our study, which could have had an inhibitory effect on migration. This observation is supported by the lower frequency of viable cells following 660 nm irradiation in the cell viability assay.

In general, the expression of keratins in the PBM groups closely mirrored that of the control group, both at the periphery and edge of the wound. The K1/K10 pair was predominantly expressed in all groups, indicating that the keratinocytes maintained their terminal differentiation^4^ even after PBM treatment with a single wavelength. Our hypothesis was that the enhancement of cell migration would be accompanied by an increase in cells positive for K5/K14, as this pair is typically more expressed in migrating basal keratinocytes,^10^, ^11^ while K1/K10 cells are associated with a stationary and well‐differentiated state.^3^ Additionally, previous studies showed that PBM using isolated red and infrared wavelengths induces strong K14 expression in epithelial projections during wound reepithelialization.^16^, ^17^ The seemingly contradictory trend between keratin expression and cell migration found in the current study may be attributed to the use of well‐differentiated immortalized cells, without any additional stimuli for dedifferentiation apart from the disruption of the cell monolayer.

Even though there was no significant increase in K5/K14 expression after PBM performed with one wavelength, the Simultaneous group showed a decrease in K1/K10 expression at the wound border compared with the control group. Additionally, there was an increase in the frequency of K5/K14‐positive cells in the wound periphery. These results indicate that simultaneous irradiation led to alterations in the basal keratin expression in migrating keratinocytes, potentially contributing to the observed reduction in wound area. Thus, the study's hypothesis that PBM using simultaneous wavelengths could enhance keratinocyte migration and alter keratin expression was supported.

This study is limited by only analyzing two pairs of keratins in relation to cell migration. The process involves cell detachment from desmosomes and hemidesmosomes, as well as changes in cytoskeleton conformation,^3^ which were not investigated in this study. Another limitation was the lack of analysis of keratinocyte migration in a stimulatory environment, such as one with the presence of cytokines and growth factors secreted by fibroblasts and other cells. We used a basic single‐cell culture model, which restricts the generalizability of the results to clinical reepithelialization scenarios. Additionally, other light parameters, such as lower time irradiation or energy density, were not tested. Based on the promising results seen in cell migration, future research could explore different light parameters to enhance the application of PBM with simultaneous irradiation for wound tissue repair.

In conclusion, the combined irradiation resulted in an enhancement of cell viability and cell migration, mainly when compared to those induced by 660 nm. This stimulation of cell migration was associated with a decrease in K1/K10 expression and an increase in K5/K14 expression, suggesting a shift in keratinocyte differentiation.

## Data Availability

The data that support the findings of this study are available from the corresponding author upon reasonable request.
